# Acidosis at Diagnosis of Type 1 Diabetes Mellitus: Relation With Kidney Function

**DOI:** 10.1155/pedi/3188571

**Published:** 2025-11-17

**Authors:** Stefano Guarino, Dario Iafusco, Anna Di Sessa, Paola Tirelli, Giulio Rivetti, Giorgia Ippolito, Mario Bartiromo, Grazia Cirillo, Angela Zanfardino, Emanuele Miraglia del Giudice, Pierluigi Marzuillo

**Affiliations:** ^1^UOC di Pediatria Generale e Specialistica, Azienda Ospedaliera Universitaria Università degli Studi della Campania “Luigi Vanvitelli”, Naples, Italy; ^2^Department of Woman, Child and of General and Specialized Surgery, Università degli Studi della Campania “Luigi Vanvitelli”, Naples, Italy

**Keywords:** acidosis, acute kidney injury, bicarbonates, ketones, proximal renal tubular dysfunction, type 1 diabetes mellitus

## Abstract

**Aims:**

Acidosis at type 1 diabetes mellitus (T1DM) onset results from unregulated massive overproduction of ketoacids. However, renal tubular damage (RTD), a complication of T1DM onset, may impair bicarbonate reabsorption, exacerbating acidosis. We aimed to assess RTD role in acidosis at T1DM onset.

**Methods:**

RTD was defined by urinary β2-microglobulin > 0.33 mg/L and/or neutrophil gelatinase-associated lipocalin (NGAL) > 95^th^ percentile for age. Acute kidney injury (AKI) was classified using Kidney Disease/Improving Global Outcomes (KDIGO) criteria. Participants were grouped by serum ketone levels (cut-off: 3 mmol/L, above which indicates diabetic ketoacidosis [DKA]) and bicarbonate levels (cut-off: 22 mmol/L, below which indicates acidosis):

– Group 1: Ketones ≥ 3 mmol/L, bicarbonate < 22 mmol/L.
– Group 2: Ketones < 3 mmol/L, bicarbonate < 22 mmol/L.
– Group 3: Ketones ≥ 3 mmol/L, bicarbonate ≥ 22 mmol/L.
– Group 4: Ketones < 3 mmol/L, bicarbonate ≥ 22 mmol/L.

**Results:**

Of 185 individuals, 111 (60%) were in Group 1, 18 (9.7%) in Group 2, 8 (4.3%) in Group 3, and 48 (26%) in Group 4. Group 1 had the most severe clinical and biochemical derangements, followed by Groups 2, 3, and 4. Logistic regression, adjusted for AKI, relative difference of weight loss and glycated hemoglobin (HbA1c), identified RTD (odds ratio [OR] = 22.3; 95% confidence interval [CI]: 6.9–71.5; *p*  < 0.001), and relative difference of weight loss, (OR = 1.2; 95% CI: 1.1–1.4; *p*  < 0.006), as significant factors associated with Group 1 and only RTD (OR = 29.9; 95% CI: 3.0–292.9; *p*=0.004) as significant factor associated with Group 2. Serum bicarbonate and blood pH showed an inverse correlation with urinary NGAL (*r*^2^ = 0.61 and 0.56, respectively, both *p*  < 0.001) and β2-microglobulin (*r*^2^ = 0.67 and 0.59, respectively, both *p*  < 0.001), regardless of ketone levels. The ketone-to-bicarbonate ratio predicted RTD (area under the receiver-operating characteristic (ROC) curve (AUROC) = 0.94; 95% CI: 0.91–0.97; *p*  < 0.001), while serum bicarbonate levels predicted normal renal tubular function (AUROC = 0.95; 95% CI: 0.92–0.98; *p*  < 0.001).

**Conclusions:**

A link exists between biological markers of RTD and metabolic acidosis at T1DM onset.

## 1. Introduction

The pathophysiological mechanism underlying metabolic acidosis in diabetic ketoacidosis (DKA) is attributed to the accumulation of ketone bodies—mainly β-hydroxybutyric acid and acetoacetic acid—resulting from absolute or relative insulin deficiency, along with elevated levels of counterregulatory hormones (particularly glucagon) and proinflammatory cytokines [1, 2].

Recent evidence suggests that at the onset of type 1 diabetes mellitus (T1DM), the kidneys may be affected by acute kidney injury (AKI) in about 40%–60% of the case [3–7] and by renal tubular damage (RTD) in about 70% of the cases at the time of clinical presentation (or diagnosis) [4]. This condition may lead to a transient Fanconi syndrome, characterized by impaired reabsorption of multiple solutes in the proximal tubule and consequent solute loss in the urine [4, 8, 9].

Proximal renal tubular cells play a pivotal role in maintaining systemic acid–base balance by reabsorbing approximately 80% of the filtered bicarbonate from the glomerulus [10, 11]. We hypothesized that RTD, which can complicate the onset of T1DM, impairs renal tubular bicarbonate reabsorption, compromising the kidney compensatory mechanism against acidosis caused by unregulated massive overproduction of ketoacids and negatively impacting clinical conditions of patients by worsening the acidosis. Furthermore, we hypothesized that the ratio between serum ketone and bicarbonate levels could help identify patients with impaired compensatory mechanisms and that normal serum bicarbonates levels could indicate normal renal tubular function.

Our aim was to analyze the role of RTD in the development of acidosis at the onset of T1DM in the Diabetes and Acute KIdney injury (DiAKIdney) cohort [4].

## 2. Materials and Methods

The DiAKIdney cohort was enrolled prospectively and consecutively between December 2017 and August 2019 [4]. The study received approval from the Research Ethics Committee of Università degli Studi della Campania “Luigi Vanvitelli” (Approval Number 368), and written informed consent was obtained from all parents. As previously outlined [4], participants were eligible if they had developed T1DM before the age of 18 and were receiving only intravenous 0.9% NaCl infusion prior to enrollment. Participants were excluded if they had been on medication for chronic conditions before the onset of T1DM, failed to attend scheduled follow-ups, or had congenital anomalies of the kidney or urinary tract.

All participants were diagnosed with autoimmune T1DM, confirmed by the presence of glutamic acid decarboxylase, islet antigen 2, insulin, and/or zinc transporter 8 antibodies at diagnosis.

### 2.1. Data Collection

After discharge, participants were followed up at 14 days. Those who had not recovered from AKI and RTD by that time returned for follow-up at 30 days and, if necessary, at 60 days.

At each evaluation, we collected data on full blood count, creatinine, glycemia, ketones (blood β-hydroxybutyrate), sodium, chloride, phosphorus, blood pH, bicarbonates, glycated hemoglobin (HbA1c) from venous blood samples, as well as urinary proteins, microalbuminuria, creatinine, sodium, calcium, phosphate, β2-microglobulin, and neutrophil gelatinase-associated lipocalin (NGAL) from urine samples. Blood pH, bicarbonates, and ketones were only assessed during hospitalization at the onset of T1DM.

### 2.2. Definitions

Dehydration was defined by the relative difference of weight loss. For all participants, weight was recorded both at T1DM onset and after 14 days, once the acute phase had resolved. The relative difference of weight loss was then calculated using the following formula: (weight after 14 days – weight at T1DM onset)/weight after 14 days.

AKI was defined according to the Kidney Disease/Improving Global Outcomes (KDIGO) criteria for serum creatinine and/or urine output [12].

From a clinical and pathophysiological point of view, it is not possible to completely differentiate tubular and glomerular involvement in AKI because they represent a continuum [13]. The initial event in AKI is tubular injury, which triggers an adaptive reduction in glomerular filtration rate through renal vasoconstriction—an attempt to limit further solute loss due to impaired tubular reabsorption [14]. If ischemia persists, this process progresses to acute tubular necrosis, shifting AKI from a functional to an intrinsic form [15, 16]. Importantly, although both NGAL and β2-microglobulin are established biomarkers of AKI, they primarily reflect tubular stress and injury [17, 18]. NGAL is rapidly released by injured tubular epithelial cells in response to ischemic or toxic damage [19], while β2-microglobulin appears in the urine when proximal tubular reabsorption is impaired [20]. Therefore, their elevation provides specific insight into tubular involvement, even within the broader context of AKI [17, 18].

However, there is no single universal cut-off for diagnosing RTD using NGAL and β2-microglobulin, and no specific expert recommendations or consensus exist for defining tubular damage in the context of AKI or at the onset of T1DM.

To ensure consistency with DiAKIdney study [4], RTD was defined by the presence of abnormal urinary β2-microglobulin (> 0.33 mg/L) and/or NGAL levels (values > 95^th^ percentile for age) [21]. Unlike the previous study [4], we did not include fractional sodium excretion (FENa) or tubular phosphate reabsorption (TRP) in the definition of RTD, aiming to obtain more precise data on tubular involvement by focusing on direct markers of tubular damage rather than calculated ones. This approach was chosen to better correlate acidosis with direct markers of tubular injury. Additionally, as in serum, glucose may interfere with urinary creatinine measurement [22], and glycosuria at T1DM onset could bias the calculation of FENa and TRP.

Because the significance of β2-microglobulin in this study differs from the previous one [4], we adjusted our approach accordingly. Previously, when β2-microglobulin levels exceeded 4 mg/L, we did not perform dilutions, as we only needed to determine the presence or absence of tubular injury. In this study, however, we remeasured β2-microglobulin in urine samples stored at – 80°C from participants with values > 4 mg/L, using dilutions to obtain precise concentration measurements.

### 2.3. Participants' Classification

To investigate the role of the kidney in acidosis at T1DM onset, we divided the population into four groups based on serum ketone and serum bicarbonate levels. We selected a cut-off of 3 mmol/L for serum ketone levels, as this is the lower threshold for defining DKA [1, 2] and a cut-off of 22 mmol/L for serum bicarbonate levels, as this represents the lower limit of normal bicarbonates in venous blood [23, 24].

**Figure 1:**
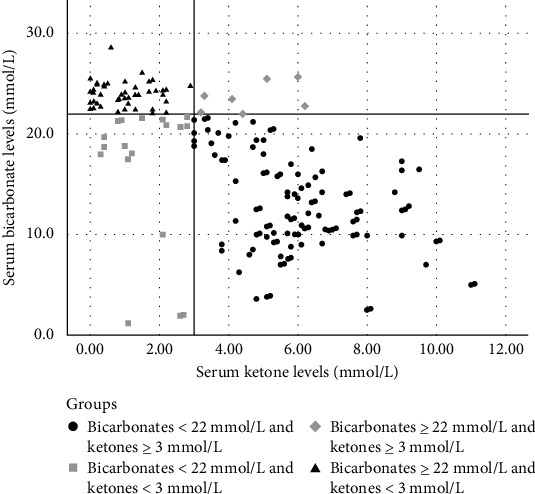
Scatter plot illustrating the distribution of the population classified by serum bicarbonate and serum ketone levels. 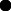
Group 1; 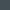
Group 2; 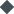
Group 3; and 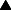
Group 4.

Therefore, the population was divided into four groups (Figure 1):

• Group 1 (acidosis and high ketones): Individuals with serum ketone levels ≥ 3 mmol/L and serum bicarbonates levels < 22 mmol/L.
• Group 2 (acidosis and low ketones): Individuals with serum ketone levels < 3 mmol/L and serum bicarbonates levels < 22 mmol/L.
• Group 3 (absence of acidosis and high ketones): Individuals with serum ketone levels ≥ 3 mmol/L and serum bicarbonates levels ≥ 22 mmol/L.
• Group 4 (absence of acidosis and low ketones): Individuals with serum ketone levels < 3 mmol/L and serum bicarbonates levels ≥ 22 mmol/L.

### 2.4. Statistical Analysis

Differences for continuous variables were analyzed with independent-sample *t*-test for normally distributed variables and with Kruskal–Wallis test in case of non-normality. The qualitative variables were compared using the chi-squared test or Fisher's exact test, as appropriate.

A scatter plot was used to illustrate the distribution of the population classified by serum bicarbonate and serum ketone levels.

Univariate and multiple logistic regression analyses were used to investigate the factors associated with Group 1 (acidosis and high ketones) and Group 2 (acidosis and low ketones) compared to Groups 3 and 4. The predictors included in these models were defined a priori based on pathophysiological mechanisms present at T1DM onset that could influence the development of acidosis, namely dehydration, kidney damage, and duration of hyperglycemia (used as surrogate marker of insulin deficiency severity). Therefore, we examined the following predictors: AKI and RTD (binary), and relative difference of weight loss and HbA1c (continuous).

All variables with *p* ≤ 0.05 were included in the multiple analysis.

A linear regression analysis was performed to assess the relationship between serum bicarbonate levels and blood pH with both urinary NGAL and urinary β2-microglobulin, stratified by the presence or absence of serum ketone levels < 3 mmol/L.

The ketone-to-bicarbonate ratio and serum bicarbonates levels were evaluated as potential predictors of RTD and normal renal tubular function, respectively, using receiver-operating characteristic (ROC) curves analysis. The Youden index was used to determine the optimal cut-off.

All statistical analyses were conducted using IBM SPSS Statistics, version 29.0.2.0.

## 3. Results

Among the 185 enrolled participants (mean age 9.1 ± 4.1 years), 129 presented with acidosis, of whom 105 had RTD. The mean time to acidosis resolution was 1.9 days (0.87 SDS). Specifically, acidosis resolved after a mean of 1.1 days (0.4 SDS) in subjects without RTD and in 2.1 (0.8 SDS) in those with RTD (*p*  < 0.001). The mean glycemia at admission of the overall population was 359.6 ± 157.6 mg/dL. The general characteristics of the population, categorized into the four groups, are presented in Table 1.

A total of 111 (60%) participants were in Group 1, 18 (9.7%) in Group 2, 8 (4.3%) in Group 3, and 48 (26%) in Group 4 (Figure 1). From Group 1 to Group 4, the severity of T1DM presentation (based on clinical and biochemical parameters) progressively decreased (Table 1).

In univariate analysis, AKI, RTD, relative difference of weight loss, and HbA1c were all significantly associated with Group 1. However, in multiple logistic regression, only RTD and relative difference of weight loss remained significantly associated (Table 2).

Conversely, in the analysis of factors associated with Group 2, univariate analysis showed a significant association with AKI, relative difference of weight loss, and RTD. In multiple analysis, only RTD retained a significant association (Table 2).

**Figure 2:**
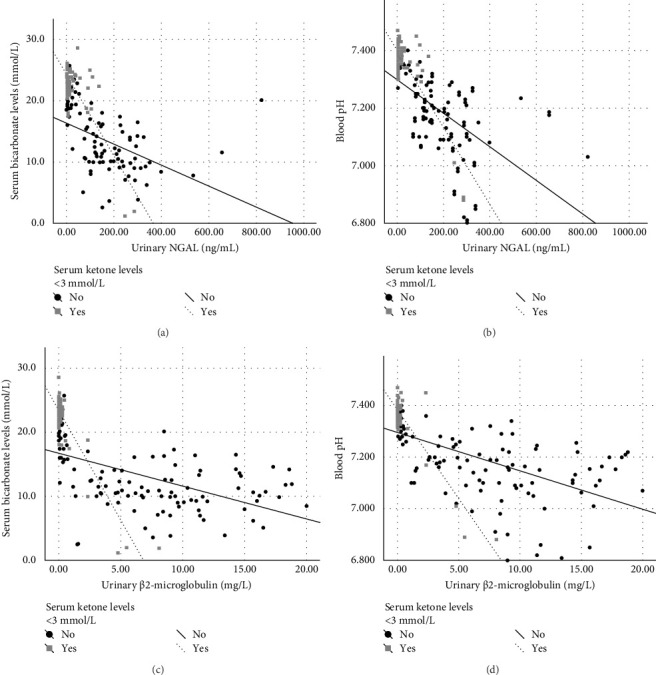
Linear regression analysis illustrating the relationship between serum bicarbonate levels and blood pH with both urinary NGAL and urinary β2-microglobulin, stratified by the presence or absence of serum ketone levels < 3 mmol/L. Panel A, relationship between serum bicarbonates levels and urinary NGAL. The regression is described by the equation *y* = 16.3187 – 0.0170859 *⁣*^*∗*^*x* + 8.00435 *⁣*^*∗*^ (serum ketone levels < 3 mmol/L, yes) − 0.0493915 *⁣*^*∗*^*x⁣*^*∗*^ (serum ketone levels < 3 mmol/L, yes). Model r2 is 0.61; *p* < 0.001. *p*-Values for intercepts and slopes are < 0.001. Panel B, relationship between blood pH and urinary NGAL. The regression is described by the equation *y* = 7.2987 – 0.000582915 *⁣*^*∗*^*x* + 0.102883 *⁣*^*∗*^ (serum ketone levels < 3 mmol/L, yes) − 0.000759809 *⁣*^*∗*^*x⁣*^*∗*^ (serum ketone levels < 3 mmol/L, yes). Model r2 is 0.56; *p* < 0.001. *p*-Values for intercepts and slopes are < 0.001. Panel C, relationship between serum bicarbonate levels and urinaryβ2-microglobulin. The regression is described by the equation *y* = 16.715 – 0.512673 *⁣*^*∗*^*x* + 6.7363 *⁣*^*∗*^ (serum ketone levels < 3 mmol/L, yes) − 2.91101 *⁣*^*∗*^*x⁣*^*∗*^ (serum ketone levels < 3 mmol/L, yes). Model r2 is 0.67; *p* < 0.001. *p*-Values for intercepts and slopes are < 0.001. Panel D, relationship between blood pH and urinary β2-microglobulin. The regression is described by the equation *y* = 7.29519 – 0.0148095 *⁣*^*∗*^*x* + 0.0887147 *⁣*^*∗*^ (serum ketone levels < 3 mmol/L, yes) − 0.0541866 *⁣*^*∗*^*x⁣*^*∗*^ (serum ketone levels < 3 mmol/L, yes). Model r2 is 0.59; *p* < 0.001. *p*-Value for intercept is 0.002 and for slopes is < 0.001.

When comparing individuals with and without serum ketone levels < 3 mmol/L, lower serum bicarbonate levels were correlated with higher urinary NGAL (Figure 2A) and β2-microglobulin (Figure 2C) levels in both groups. Similarly, a comparable relationship was observed between blood pH and both urinary NGAL (Figure 2B) and β2-microglobulin (Figure 2D).

**Figure 3:**
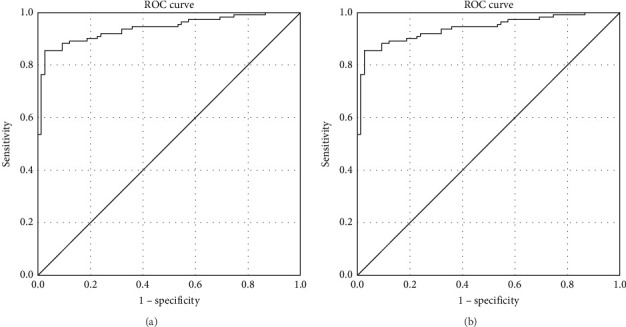
ROC curve analysis of the ketone-to-bicarbonate ratio as a predictor of RTD (Panel A) and of serum bicarbonates levels as predictor of normal renal tubular function (Panel B).

Evaluating the ketone-to-bicarbonate ratio as predictor of RTD by ROC curve analysis, the area under the ROC curve (AUROC) was 0.94 (95% confidence interval [CI]: 0.91–0.97; *p*  < 0.001) (Figure 3A). Based on the Youden index, the optimal cut-off for the ketone-to-bicarbonate ratio was ≥ 0.27, predicting RTD with a sensitivity of 85.5% and a specificity of 97.3%. On the other hand, evaluating serum bicarbonate levels as predictors of normal renal tubular function, the AUROC was 0.95 (95% CI: 0.92–0.98; *p*  < 0.001) (Figure 3B). The optimal cut-off for serum bicarbonates levels was ≥ 19.65 mmol/L, predicting a normal tubular function with a sensitivity of 90.7% and a specificity of 92.7%.

## 4. Discussion

Our findings indicate that RTD, associated with the onset of T1DM, worsens the severity of metabolic acidosis caused by the unregulated overproduction of ketoacids [1, 2]. We showed that not all children and adolescents with acidosis at T1DM onset present with DKA. In fact, 9.7% of individuals (Group 2) had acidosis but serum ketone levels below 3 mmol/L, a threshold considered a sensitive indicator of DKA [1, 2]. Notably, this group comprises two markedly different profiles: one characterized by mild acidosis (bicarbonate > 18 mmol/L) and the other by severe acidosis, including three subjects with bicarbonate levels near the detection limit.

This could indicate the existence of mild metabolic ketoacidosis without increased anion gap, most likely due to tubular dysfunction at the onset of T1DM.

Analyzing potential factors associated with this condition, including dehydration, AKI, RTD, and HbA1c, RTD remained the only determinant after adjustments (Table 2). Furthermore, when assessing these factors in relation to acidosis and high serum ketone levels (Group 1), RTD emerged once again as a significant factor along with relative difference of weight loss in multiple logistic regression (Table 2).

These findings are further supported by regression analyses, which revealed a strong inverse relationship between serum bicarbonate levels or blood pH and urinary NGAL or β2-microglobulin in both participants with serum ketone levels < 3 mmol/L and those with serum ketone levels ≥ 3 mmol/L (Figure 2). According to the hypothesis behind our study, this indicates that, in addition to ketoacid accumulation, RTD plays a pivotal role in determining acidosis at T1DM onset. Indeed, regardless of serum ketone levels, acidosis is directly linked to biomarkers of tubular damage. Moreover, the presence of RTD was associated with a delayed recovery from acidosis.

Interestingly, individuals with high serum ketone levels but without acidosis (Group 3) had a lower prevalence of RTD and lower levels of NGAL and β2-microglobulin compared to Groups 1 and 2. More specifically, in Group 3, only one patient met the RTD criteria and showed only a very mild increase in β2-microglobulin levels. This suggests that when serum ketone levels are high, in the presence of normal renal tubular function, homeostasis is maintained.

However, when the T1DM presentation is severe, more ketoacids accumulate, and simultaneously, RTD contributes to the severity of acidosis. In fact, we found that children presenting with acidosis and high serum ketone levels (Group 1) had the worst clinical and metabolic conditions, followed by those with acidosis and low serum ketone levels (Group 2). These children were more severely affected than participants with high serum ketone levels but without acidosis (Group 3), who, in turn, were more affected than individuals with low ketone levels and no acidosis (Group 4).

Therefore, the severity of T1DM onset influences the development of both AKI and RTD, which are closely interconnected [3–6, 8, 9]. In turn, RTD exacerbates acidosis by disrupting the kidney reabsorption of bicarbonates.

Furthermore, the pronounced volume depletion, systemic hypotension, and vasoconstriction associated with both DKA and its accompanying metabolic acidosis could critically compromise kidney perfusion [8, 9, 25, 26]. These severe hemodynamic changes can precipitate AKI—a complication that, by exacerbating tubular dysfunction, not only prolongs the resolution of the acidosis but also represents a significant risk factor contributing to the development of chronic kidney disease later in life [27].

Although tubular injury at T1DM onset has been previously described [4, 8, 9], the direct association with acidosis severity has not been systematically investigated before. However, Tinti et al [28]. described three children in which T1DM onset was characterized by severe acidosis (pH < 7.1) in the absence of DKA. Interestingly, all three subjects developed AKI. Although tubular function was not assessed [28], our findings could suggest that RTD associated with AKI may have contributed to low bicarbonate levels, thereby exacerbating the severity of acidosis despite low levels of ketone bodies.

As a future perspective, it would be interesting to investigate the underlying mechanisms of tubular injury itself (e.g., glucotoxicity, hypovolemia, inflammatory mediators, etc.).

A limitation of our study is the single-center enrollment. However, the prospective design, the standardized biochemical measurements performed in the same laboratory, and the availability of direct markers of tubular damage, such as NGAL and β2-microglobulin, could partially mitigate this limitation. Another limitation is the unequal distribution of participants across the four groups, with Group 3 including only eight participants. However, this distribution reflects what typically occurs in a population of children at the onset of T1DM. Nevertheless, significant associations were observed, and the association between RTD and acidosis was confirmed in the overall population using linear regression.

To estimate weight loss as a measure of dehydration, we used the weight at the first follow-up (+ 14 days) as the baseline value. We acknowledge that part of this weight gain was attributable to the anabolic effect of insulin; however, we believe that within only 14 days this effect primarily contributed to restoring baseline weight rather than leading to excess weight. Notably, this method of calculating weight loss in these subjects correlated well with heart rate variation relative to baseline, which is considered a reliable biomarker of dehydration [29].

Finally, chloride and bicarbonate have an inverse relationship in the body. When chloride is lost, the body compensates by increasing bicarbonate reabsorption to maintain electrical balance [30]. The effect of extracellular volume expansion was investigated in dogs receiving isotonic saline, and it was found that extracellular volume expansion suppressed bicarbonate reabsorption [31]. Therefore, chloride infusion could influence bicarbonate reabsorption.

According to our healthcare organization, all patients with new-onset T1DM are transferred from local referral centers within 2 h of admission. Among enrolled participants, 85% were transferred within 60 min, 10% within 90 min, and 5% within 120 min (median: 40 min) [4]. During transfer, all individuals received an infusion of 0.9% NaCl. Thus, at the time of our assessment, all individuals had undergone the same procedure (infusion of 0.9% NaCl only) and for this reason we did not consider the chloride as confounder. However, considering that the saline infusion is linked to the dehydration degree, logistic regression analyses considered the degree of dehydration in the multiple logistic regression.

## 5. Conclusions

In conclusion, we showed that the kidneys play a significant role in exacerbating acidosis at the onset of T1DM. RTD, closely associated with T1DM onset, may impair tubular mechanisms of acid–base compensation, thereby contributing to the worsening of the acidotic state.

## Figures and Tables

**Table 1:** Clinical characteristics of the study population divided into four groups based on serum bicarbonate and ketone levels.

Parameters and derived classifications		Group 1 (*n* = 111)	Group 2 (*n* = 18)	Group 3 (*n* = 8)	Group 4 (*n* = 48)	Global *p*
Clinical parameters and derived classifications	Age (year), mean (SDS)	9.0 (4.2)	7.9 (4.0)	9.7 (4.5)	9.6 (3.5)	0.45
	Male sex, No. (%)	43 (38.7)	10 (55.6)	4 (50.0)	24 (50.0)	0.38
	Relative difference of weight loss (%), mean (SDS)	9.6 (6.1)	9.1 (11.7)	4.8 (4.1)	3.7 (3.4)	<0.001
	Relative difference of weight loss > 5%, No. (%)	85 (76.6)	9 (50)	3 (37.5)	16 (33.3)	<0.001
	Relative difference of weight loss > 10%, No. (%)	42 (37.8)	6 (33.3)	1 (12.5)	3 (6.3)	<0.001
	T1DM familial history, No. (%)	7 (6.3)	2 (11.1)	2 (25.0)	14 (29.2)	0.001
	BMI (SDS), mean (SDS)	0.8 (1.7)	−1.6 (2.6)	0.08 (1.6)	−0.04 (1.1)	0.004
	SBP (SDS), mean (SDS)	0.50 (0.9)	0.74 (0.73)	0.37 (0.86)	0.62 (0.78)	0.59
	DBP (SDS), mean (SDS)	0.37 (0.85)	0.18 (0.51)	0.47 (0.48)	0.31 (0.91)	0.79
	Kussmaul breathing, No. (%)	25 (22.5)	4 (22.2)	0 (0)	0 (0)	0.002
	Coma^a^, No. (%)	20 (18)	2 (16.7)	0 (0)	0 (0)	0.01

Biochemical parameters and derived classifications	Glucose, mg/dL, mean (SDS)	394.6 (161.9)	385.5 (122.4)	388.7 (100.0)	264.0 (128.0)	<0.001
	pH, mean (SDS)	7.18 (0.14)	7.27 (0.17)	7.37 (0.02)	7.38 (0.04)	<0.001
	Serum bicarbonate level (mEq/L), mean (SDS)	12.7 (4.7)	16.4 (7.3)	23.4 (1.5)	23.9 (1.2)	<0.001
	Serum ketones (mmol/L), mean (SDS)	6.0 (1.8)	1.6 (0.9)	4.4 (1.2)	0.98 (0.78)	<0.001
	Anion Gap^b^, mean (SDS)	24.0 (7.0)	19.8 (5.3)	18.5 (4.0)	14.5 (3.5)	<0.001
	eGFR, mL/min/1.73 m^2^, mean (SDS)	87.0 (24.1)	96.6 (25.0)	118.3 (37.3)	111.1 (18.1)	<0.001
	Creatinine (mg/dL), mean (SDS)	0.91 (0.27)	0.76 (0.15)	0.73 (0.24)	0.72 (0.18)	<0.001
	Diuresis (first 6 h) (mL/kg/h), mean (SDS)	1.28 (0.65)	1.38 (0.75)	1.01 (0.53)	1.29 (0.57)	0.74
	AKI, No. (%)	67 (60.4)	7 (38.9)	2 (25.0)	5 (10.4)	<0.001
	UPr/Cr (mg/mg), median (IQR)	0.85 (1.0)	0.30 (0.46)	0.20 (0.07)	0.21 (0.14)	<0.001
	Microalbuminuria (mg/L), median (IQR)	35 (88)	8 (46)	8 (15)	12 (33)	<0.001
	Proteinuria, No. (%)	102 (91.9)	15 (83.3)	4 (50)	25 (52.1)	<0.001
	Urinary NGAL (ng/mL), median (IQR)	150.3 (181.8)	83.2 (160.7)	15.3 (21.4)	7.9 (20.3)	<0.001
	Urinary beta-2 microglobulin (mg/L), mean (SDS)	6.8 (5.7)	1.4 (2.4)	0.2 (0.14)	0.08 (0.10)	<0.001
	RTD, No. (%)	94 (84.7)	11 (61.1)	1 (12.5)	4 (8.3)	<0.001
	Corrected serum sodium level (mEq/L), median (IQR)	140.1 (4.3)	139.4 (2.6)	141.2 (1.6)	140.1 (2.4)	0.69
	Serum sodium >145 mEq/L, No. (%)	8 (7.2)	0 (0)	0 (0)	1 (2.1)	0.58
	Serum chloride levels (mEq/L), mean (SDS)	103.3 (5.0)	102.1 (5.2)	99.2 (4.1)	101.6 (2.5)	0.02
	Serum potassium (mEq/L), mean (SDS)	4.05 (0.67)	4.1 (0.4)	4.0 (0.22)	4.3 (0.55)	0.17
	Serum potassium < 3.5 mEq/L	19 (17.1)	1 (5.6)	0 (0)	0 (0)	0.003
	Serum potassium ≤ 2.5 mEq/L	3 (2.7)	0 (0)	0 (0)	0 (0)	0.72
	HbA1c (%) mean (SDS)	12.1 (1.8)	10.7 (1.4)	11.0 (1.6)	11.5 (2.0)	<0.001

Ultrasound parameters	Abnormal kidney echogenicity, No (%)	42 (37.8)	3 (16.7)	0 (0)	0 (0)	<0.001
	Renal length (SDS), mean (SDS)	1.31 (1.45)	0.96 (1.50)	1.6 (1.2)	1.1 (1.40)	0.54

*Note:* Mean and SDS are reported for normally distributed variables, whereas median and IQR range are provided for non-normally distributed variables.

Abbreviations: AKI, acute kidney injury; BMI, body mass index; DBP, diastolic blood pressure; eGFR, estimated glomerular filtration rate; Hb1Ac, glycated hemoglobin; IQR, interquartile range; NGAL, neutrophil gelatinase-associated lipocalin; No., number; RTD, renal tubular damage; SBP, systolic blood pressure; SDS, standard deviation score; T1DM, type 1 diabetes mellitus; UPr/Cr, urinary protein-to-creatinine ratio.

^a^The level of consciousness was assessed by Glasgow coma scale. Coma was defined as a Glasgow coma score below 12 for > 6 h.

^b^According to the ISPAD clinical practice consensus guidelines, the anion gap was calculated as follows: anion gap = Na – (Cl + HCO_3_).

**Table 2:** Logistic regression analysis investigating factors associated with Group 1 (acidosis and high ketones) and Group 2 (acidosis and low ketones).

Investigated factors	Group 1^a^				Group 2^a^			
	Univariate		Multiple		Univariate		Multiple	
	OR (95% CI)	*p*	OR (95% CI)	*p*	OR (95% CI)	*p*	OR (95% CI)	*p*
AKI	10.9 (4.5–26.2)	<0.001	2.5 (0.7–8.2)	0.14	4.5 (1.3–15.3)	0.018	3.9 (0.3–45.3)	0.28
RTD	56.4 (19.7–161.8)	<0.001	22.3 (6.9–71.5)	<0.001	16 (4.2–59.9)	<0.001	29.9 (3.0–292.9)	0.004
Relative difference of weight loss (%)	1.31 (1.2–1.5)	<0.001	1.2 (1.1–1.4)	0.006	1.1 (1.02–1.2)	0.023	1.1 (0.96–1.2)	0.20
Hb1Ac (%)	1.6 (1.3–1.9)	<0.001	1.2 (0.9–1.7)	0.16	1.1 (0.8–1.4)	0.66	–	–

Abbreviations: AKI, acute kidney injury; CI, confidence interval; Hb1Ac, glycated hemoglobin; OR, odds ratio; RTD, renal tubular damage.

^a^Compared to Groups 3 and 4.

## Data Availability

The datasets used and/or analyzed during the current study are available from the corresponding author on reasonable request.
